# Single-Cell Whole-Transcriptome Detection Expands the Observable RNA Space for AI Virtual-Cell Models

**DOI:** 10.3390/bios16100581

**Published:** 2026-10-16

**Authors:** Xudong Liu, Bo Zhang, Kefei Zou, Ruikai Wan, Qiangyuan Zhu

**Affiliations:** State Key Laboratory of Industrial Control Technology, Research Center for Analytical Instrumentation, Institute of Cyber-Systems and Control, College of Control Science and Engineering, Zhejiang University, Hangzhou 310000, China; lxd112@zju.edu.cn (X.L.); 22432117@zju.edu.cn (B.Z.); kfzou@zju.edu.cn (K.Z.); 22532027@zju.edu.cn (R.W.)

**Keywords:** single-cell biosensing, single-cell whole-transcriptome detection, AI-enabled virtual-cell models, RNA regulatory networks, predictive modeling

## Abstract

Single-cell RNA sequencing has supplied cell atlases and artificial intelligence (AI)-enabled virtual-cell models with scalable, gene-level descriptions of cellular state. Yet prevalent poly(A)-based counting assays capture only part of the RNA landscape. Here, “single-cell whole-transcriptome detection” denotes sequencing-based efforts to detect a broader range of RNA biotypes than conventional poly(A)-based gene counting; no assay detects or quantifies every cellular RNA species without bias. We compare RNA-capture scope, transcript-structure resolution, temporal labeling, spatial context, and additional modalities as distinct measurement dimensions. From a biosensing perspective, analytical sensitivity, transcript coverage, throughput, and assay bias determine which signals are available to computational models. We then examine cell-state representations, regulatory networks, dynamics, perturbation prediction, and foundation models. Total-RNA assays reveal additional non-coding RNAs, long-read methods inform transcript structure, and temporal assays constrain RNA kinetics. Whether these signals improve prediction beyond conventional gene-count inputs requires matched, task-specific model comparisons. We distinguish molecular observability, statistical association, predictive performance, and causal validation, which requires appropriate interventions. We propose benchmarks controlling sequencing depth, cost, model architecture, and held-out samples while testing feature ablations, out-of-distribution performance, uncertainty calibration, and experimental outcomes. Together, high-throughput poly(A)-based profiling and broader RNA measurements provide complementary cellular breadth and regulatory depth.

## 1. Introduction

Cells link genotype and molecular regulation to tissue function and disease, yet bulk RNA sequencing averages signals across heterogeneous populations. Single-cell profiling has made cellular identity and state measurable at much finer resolution. The Human Cell Atlas (HCA), launched as an international effort and articulated in its 2017 white paper, aims to map human cells across tissues and contexts using shared standards for measurement, annotation, and integration [1]. Studies have shown how cellular identities and aging-associated programs can be compared across mouse organs and ages [2,3]. Tabula Sapiens extended this atlas approach across human tissues, while a human fetal chromatin atlas connected cell identities to candidate regulatory elements [4,5]. Together, these resources provide references of observed states; they do not themselves predict how an unseen intervention will change a cell.

The idea of a virtual cell predates modern artificial intelligence (AI). The Virtual Cell framework modeled biochemical reactions and spatial dynamics, and an integrated model of *Mycoplasma genitalium* linked multiple cellular processes to simulated phenotypes [6,7]. Extending such mechanistic models to mammalian systems is difficult because many interactions, kinetic parameters, and context-dependent cell states remain unknown. More recent proposals outline how AI might build transferable representations and experimentally testable predictions from multiscale data [8]. A CM4AI preprint describes an initiative to generate AI-ready maps of human cell architecture from disease-relevant cell lines [9]. Mechanistic and data-driven approaches need not be mutually exclusive. In either case, a useful virtual-cell model must move beyond cell labeling to predict state changes under specified conditions.

Single-cell RNA sequencing (scRNA-seq) has become a principal measurement layer for this goal. Early assays established cell-resolved transcript profiling [10]. Droplet barcoding and unique molecular identifiers (UMIs) subsequently enabled much larger cell atlases [11,12]. These data support latent-state models such as scVI, genetic-perturbation readouts such as Perturb-seq, and assumption-dependent estimates of state-transition direction from unspliced and spliced RNA [13,14,15]. They also provide large training corpora for foundation models, including Geneformer and scGPT [16,17]. However, the information available to these models is constrained by the capture and readout properties of the assays that generated their training data.

Many large scRNA-seq datasets comprise gene-level counts from poly(A)-based end-counting workflows. Such measurements are effective for mapping cellular identity and state but provide limited access to many non-polyadenylated RNAs, transcript structures, and RNA-processing intermediates. Here, “the whole transcriptome” denotes the full repertoire of RNA molecules present in a cell at a defined time, regardless of poly(A) status. We use “single-cell whole-transcriptome detection” to describe sequencing-based efforts to detect components of this repertoire across RNA biotypes, particularly through total-RNA capture. The term indicates the scope of the biological target, not complete or unbiased recovery of every RNA species by a single assay. Total-RNA capture, transcript-structure analysis, and temporal RNA labeling provide different, sometimes complementary forms of RNA information. Spatial RNA profiling and multi-omics add complementary positional and non-RNA information; these capabilities are not inherent to single-cell whole-transcriptome detection. From a biosensing perspective, capture efficiency, detection sensitivity, the fraction of informative reads, sequencing saturation, and cellular throughput determine which molecular signals can enter a computational model. Total-RNA methods such as VASA-seq have demonstrated broader RNA detection and gene-body coverage than conventional end-counting assays [18], while a Tabula Sapiens total-RNA preprint reports cell-type-specific non-coding RNA patterns across human tissues [19]. These studies establish additional observables; whether those observables improve AI prediction remains a separate question.

We therefore examine how assay design defines measurable RNA signals and how those signals enter virtual-cell models. We first compare capture breadth, transcript-structure resolution, temporal information, spatial context, added modalities, and analytical performance. We then review cell-state representations, regulatory networks, dynamics, perturbation models, and foundation models. Throughout, we distinguish what is measured, what is statistically associated, what predicts unseen data, what supports a mechanistic interpretation, and what has been causally validated. These are distinct levels of evidence, not an automatic progression. Finally, we propose matched benchmarks to test whether additional RNA variables improve specified predictions in unseen biological contexts.

This narrative review is organized around single-cell measurement capabilities and virtual-cell modeling questions. Candidate literature was assembled iteratively during manuscript preparation, drawing on primary assay and modeling studies and selected conceptual reviews that frame virtual-cell applications. We selected representative primary studies for the distinct assay capabilities or modeling approaches they introduced and for their relevance to measurement-to-model evaluation. Where available, we give particular attention to direct assay comparisons and experimentally evaluated model outputs. We distinguish peer-reviewed studies from preprints and identify developer-reported performance comparisons as such. Study selection was not based on a prespecified database search or formal screening protocol, and no fixed search cutoff was used; accordingly, this review does not claim exhaustive literature coverage.

## 2. Evolution of Single-Cell Transcriptomic Measurement Technologies: From Poly(A) RNA to a Broader Observable RNA State Space

Single-cell transcriptomic assays can be viewed as sequencing-enabled molecular biosensing workflows. Cell or nucleus isolation defines the sampled biological unit, while permeabilization, lysis, capture chemistry, reverse transcription, depletion, amplification, and barcoding determine which RNA molecules contribute to the measured data. These steps create an observation process between the cellular RNA state and the inputs available to virtual-cell models. Comparing assays therefore requires more than listing the RNA classes they target: relevant analytical properties include RNA-biotype-specific detection limits, capture efficiency and quantitative dynamic range, library complexity, the fraction of usable reads not derived from ribosomal RNA (rRNA), gene-body and strand coverage, molecular counting accuracy, background and false-positive signals, cell recovery, sequencing saturation, throughput, assay time, and cost at a stated sequencing depth [20]. Because these properties also depend on sample type and data processing, nominal RNA coverage does not necessarily translate into equally informative model inputs. A transcript recovered only sporadically, or only with substantially greater sequencing investment, may offer limited value for a given modeling task.

Single-cell RNA technologies differ along several partly independent axes. Capture chemistry determines which RNA biotypes enter the library; read distribution and read length shape gene-abundance and splice-junction measurements, while full-length isoform reconstruction also requires recovery of transcript-spanning molecules; metabolic labeling provides temporal information; spatial assays preserve tissue coordinates; and multi-omics assays add non-RNA measurements. Cellular throughput and molecular sensitivity vary across implementations and sequencing budgets. We therefore compare these properties separately rather than treating ‘whole-transcriptome’ as a single performance class.

### 2.1. Low-Throughput Poly(A)-Based RNA Sequencing

Low-throughput poly(A)-based RNA sequencing encompasses early single-cell messenger RNA (mRNA) sequencing protocols and plate-based methods such as the Smart-seq family. In a typical workflow, individual cells are deposited into separate wells by micromanipulation, microfluidic capture, or fluorescence-activated cell sorting. Following cell lysis, polyadenylated RNAs are captured with oligo-dT primers and converted into complementary DNA (cDNA), which is subsequently amplified, fragmented, adapter-ligated, and analyzed by short-read sequencing. Because cells remain physically separated during the early stages of library construction, they can be selected according to predefined phenotypes, and information about their origin, surface markers, and experimental conditions can be retained.

The early single-cell mRNA-sequencing method developed by Tang and colleagues demonstrated that thousands of genes and numerous splice junctions could be detected from an individual cell [10]. Smart-seq uses an oligo-dT primer carrying a 5′ universal adapter to initiate reverse transcription from the poly(A) tail. When the Moloney murine leukemia virus-derived reverse transcriptase reaches the 5′ end of the RNA template, its terminal transferase activity adds several non-templated cytosines to the 3′ end of the nascent first-strand cDNA. These cytosines facilitate annealing of a template-switching oligonucleotide, which is subsequently copied by the reverse transcriptase to append a second universal sequence to the 3′ end of the cDNA. The resulting cDNA therefore contains known adapter sequences at both ends and can be amplified using universal polymerase chain reaction (PCR) primers, producing relatively broad gene-body coverage of polyadenylated transcripts [21]. Smart-seq2 optimized reverse transcription, template switching, and PCR amplification to improve sensitivity, accuracy, and coverage of longer transcripts [22]. Smart-seq3 introduced a 5′ UMI and combined UMI-containing terminal reads with internal transcript reads, enabling a subset of RNA molecules to be counted and reconstructed at allele and isoform resolution [23]. Smart-seq3xpress miniaturizes the well-based Smart-seq3 workflow and directly tagments diluted, preamplified cDNA, bypassing intermediate cDNA clean-up, concentration measurement, and fragment-length quality control while retaining 5′ UMI-containing and internal gene-body reads. In the cited study, 26,260 cells were processed in individual wells across multiple 384-well plates with automation-assisted reagent dispensing; this is the cumulative study scale, not the throughput of a single run [24]. Here, “low-throughput” describes the relative processing architecture, not a fixed upper limit on the number of cells in a study.

The principal strengths of these methods are their high information content per cell and relatively even gene-body coverage, which make them suitable for detecting low-abundance genes, alternative splicing, allele-specific expression, expressed somatic variants, and transcript isoforms. Their ability to profile prespecified cells is also valuable when target populations are rare or clinical material is limited. However, well-by-well processing and relatively deep sequencing increase the experimental and sequencing cost per cell, while cellular throughput remains lower than that of droplet-based or combinatorial-indexing methods. Moreover, “full-length” refers to broad coverage along polyadenylated transcripts; it does not imply that every RNA molecule is read from end to end or that non-polyadenylated RNAs are captured without bias.

### 2.2. High-Throughput Poly(A)-Based RNA Sequencing

High-throughput poly(A)-based RNA sequencing relies on the early assignment of barcodes to cells and RNA molecules, allowing material from many cells to be pooled for amplification and library construction. In droplet-based workflows, individual cells, lysis reagents, and barcoded capture beads are co-encapsulated in nanoliter droplets. Oligonucleotides on each bead typically contain a PCR handle, a cell barcode, a UMI, and an oligo-dT capture sequence. Following cell lysis, poly(A) RNAs hybridize to the oligo-dT sequence and acquire cell barcodes and UMIs during reverse transcription. The emulsion is then broken, barcoded cDNAs are pooled and amplified, and 3′- or 5′-end libraries are constructed.

Drop-seq and inDrop used barcoded beads or hydrogel beads to label transcripts within droplets, expanding scRNA-seq to thousands or tens of thousands of cells [11,25]. Seq-Well introduced a subnanoliter microwell array in which cells and barcoded beads are sealed with a semipermeable membrane, improving portability and compatibility with low-input samples while retaining high throughput [26]. The 10x Genomics GemCode system combined droplet generation and gel-bead barcoding for high-throughput 3′-end gene counting [12]. Combinatorial indexing provides an alternative route to high throughput. In sci-RNA-seq and SPLiT-seq, fixed and permeabilized cells or nuclei receive cell-identifying barcodes through successive split-pool rounds, without remaining in separate reaction compartments throughout library preparation. In the studies cited here, sci-RNA-seq profiled nearly 50,000 C. elegans cells [27], whereas SPLiT-seq generated 156,049 single-nucleus transcriptomes from developing mouse brain and spinal cord [28].

UMIs distinguish cDNAs derived from different RNA molecules and reduce the influence of PCR amplification bias on gene-level quantification. High cellular throughput facilitates the discovery of rare populations, construction of cross-tissue atlases, characterization of continuous cell states, and implementation of large perturbation screens such as Perturb-seq. High-throughput poly(A)-based RNA sequencing has therefore generated much of the data used in current cell atlases and single-cell foundation models. Its principal limitation is that most reads originate near the 3′ or 5′ end of each transcript, and fewer informative reads are generally obtained per cell than with plate-based protocols. The typical output is consequently a gene-level UMI count matrix, with limited ability to resolve complete isoforms, coordinated splicing events, allele-specific expression, non-polyadenylated RNAs, or short regulatory RNAs. Droplet doublets, ambient RNA contamination, tissue-dissociation bias, and cell-type-dependent capture efficiency can further distort the observed cellular state space.

### 2.3. Single-Cell Total-RNA Capture

Single-cell total-RNA capture methods seek to reduce the RNA-biotype restrictions imposed by oligo-dT-based workflows and to make non-polyadenylated RNAs, RNA precursors, splicing intermediates, and selected short non-coding RNAs experimentally observable. A central technical challenge is that each cell contains little RNA, while ribosomal RNA constitutes a large fraction of total RNA. Random priming broadens molecular capture but also generates large amounts of rRNA-derived cDNA. These methods therefore combine strategies such as random priming, enzymatic polyadenylation, RNA fragmentation, molecular barcoding, and rRNA depletion.

SUPeR-seq replaces exclusive oligo-dT priming with random primers containing fixed anchor sequences, allowing both polyadenylated and non-polyadenylated RNAs to enter the reverse-transcription and amplification workflow. It was initially applied to the analysis of linear and circular RNAs in preimplantation embryos [29]. MATQ-seq uses multiple annealing and dC tailing to introduce universal sequences into cDNA, providing highly sensitive capture from low-input total RNA [30]. RamDA-seq combines oligo-dT primers with “not-so-random” primers designed to avoid rRNA sequences and amplifies cDNA during reverse transcription through random displacement. It provides near-full-length coverage and enables the detection of non-polyadenylated histone RNAs, long non-coding RNAs (lncRNAs), enhancer RNAs, and RNA-processing events such as recursive splicing [31]. Complementing these capture chemistries, scDASH is a library-depletion module rather than an independent RNA-capture method. It uses rRNA-specific guide RNAs and Cas9 to cleave rRNA-derived cDNA, thereby increasing the fraction of informative non-rRNA reads [32].

Smart-seq-total first adds poly(A) tails to cellular RNAs enzymatically and then performs oligo-dT-primed reverse transcription. A degradable template-switching oligonucleotide is selectively removed after reverse transcription, reducing adapter-derived products that would otherwise interfere with short-RNA detection. Smart-seq-total and its UMI-compatible implementation can jointly detect protein-coding RNAs, lncRNAs, microRNAs (miRNAs), small nucleolar RNAs (snoRNAs), and other small non-coding RNAs (sncRNAs) from the same cell [33]. VASA-seq instead fragments RNA in the cell lysate, repairs and polyadenylates the resulting fragments, and performs reverse transcription using oligo-dT primers containing cell barcodes and unique fragment identifiers. Barcoded cDNA is then linearly amplified by in vitro transcription, followed by rRNA depletion. VASA-seq is compatible with both plate and droplet formats and, in more than 30,000 mouse embryonic cells, detected non-polyadenylated and non-coding RNAs, alternative splicing, and increased unspliced-RNA signal [18]. The latter supported model-dependent RNA-velocity inference, but the assay did not directly track the same cells over time or measure RNA synthesis and degradation rates without additional assumptions or temporal measurements.

A preprint describing STORM-seq reports a workflow that combines random priming, UMI-containing template switching, strand-specific library preparation, and rRNA depletion to generate single-cell total RNA libraries with broad gene-body coverage [34]. More recently, TotalX adapted enzymatic polyadenylation, a degradable template-switching oligonucleotide, and Cas9-mediated rRNA depletion to the 10x Genomics 3′ workflow. By processing long and short cDNA fractions separately, TotalX enables droplet-scale measurement of diverse coding and non-coding RNAs, with an optional procedure for enriching miRNAs [35]. A recent preprint coauthored by X.L. and Q.Z., both authors of this review, describes dropTotal, a poly(A)-independent droplet-based total-RNA workflow [36]; their related interests are disclosed in the Conflicts of Interest statement. The method combines temperature-ramped hybridization of a dU-modified reverse-transcription primer, Uracil-Specific Excision Reagent (USER)-mediated primer cleavage, and droplet-merging barcoding. The authors report strand-specific libraries and relatively even gene-body coverage. In their HEK293T downsampling comparison with VASA-drop, TotalX, MATQ-drop, and 10x Chromium, dropTotal recovered more genes at the matched read depths examined. At 50,000 reads per cell, the reported median was 1189.5 detected lncRNAs per cell, approximately twice that reported for VASA-drop under the study’s comparison conditions. These are author-reported results from a preprint; their generalizability across tissues, sample preparations, sequencing budgets, and independent laboratories remains to be established.

Together, these total-RNA methods broaden the observable RNA repertoire through different capture, amplification, and rRNA-management strategies; none provides exhaustive or unbiased recovery of all cellular RNAs. Their analytical performance depends on sample type, RNA biotype, sequencing depth, library processing, and the analysis pipeline, making sensitivity claims specific to the conditions under which they were evaluated. Broader detection of candidate non-coding RNAs can help prioritize them for follow-up experiments, but detection and association alone neither establish RNA function nor demonstrate improved virtual-cell prediction.

### 2.4. Long-Read Sequencing and Single-Cell Multi-Omics

Broader RNA capture and greater transcript-structure resolution are distinct, though potentially complementary, capabilities. Total-RNA methods expand the range of RNA molecules that can enter a library, whereas long-read sequencing provides structural information about molecules captured upstream. Temporal labeling, spatial profiling, and multi-omics add other forms of information through separate experimental steps; none follows automatically from total-RNA capture. Single-cell long-read workflows commonly sequence full-length or near-full-length cDNA generated using methods such as Smart-seq or adapted 10x Genomics protocols, rather than native cellular RNA. These cDNAs are sequenced on PacBio or Oxford Nanopore platforms. Where present, cell barcodes and UMIs are recovered and error-corrected to support the assignment of reads to individual cells. Reliable transcript-structure analysis also depends on the recovery of transcript-spanning molecules and adequate sequencing accuracy.

ScISOr-Seq combined 10x cell barcodes with PacBio long-read sequencing to characterize cell-type-specific isoforms in thousands of cerebellar cells [37]. R2C2 circularizes cDNA molecules and uses rolling-circle amplification to generate repeated copies of each template, from which more accurate Oxford Nanopore consensus sequences can be derived [38]. MAS-ISO-seq programmably concatenates cDNA molecules into constructs optimized for PacBio sequencing, increasing cDNA read recovery and improving the detection of differential splicing at single-cell resolution [39]. HIT-scISOseq enriches barcoded 10x cDNA to reduce template-switching artifacts and concatenates cDNA molecules for PacBio circular-consensus sequencing. In the authors’ comparison with ScISOr-Seq, it increased informative long-read yield [40]. Nevertheless, it inherits the upstream poly(A)-based capture spectrum and cannot recover RNA biotypes excluded during library preparation. Full-length isoform reconstruction also depends on recovery of transcript-spanning cDNA, adequate sequencing accuracy, and correct barcode assignment; molecule-length bias, limited informative reads per cell, and sequencing cost remain constraints. Short reads can therefore provide high-throughput gene-level quantification, while long reads contribute cell-type- or state-specific isoform information.

Single-cell multi-omics provides regulatory context or functional readouts by measuring RNA together with additional molecular modalities in the same cell. CITE-seq labels cell-surface proteins with DNA-barcoded antibodies; antibody-derived tags and mRNAs receive the same cell barcode during library preparation, enabling paired measurements of RNA and protein abundance [41]. sci-CAR and SHARE-seq separately label accessible chromatin fragments and RNA-derived cDNA in permeabilized cells or nuclei and use combinatorial indexing to associate both molecular classes with their cell of origin. These assays connect chromatin accessibility and candidate regulatory elements with gene expression in the same cells [42,43]. TEA-seq further combines transcript abundance, surface-protein epitopes, and chromatin accessibility, producing a trimodal readout of transcriptional state, protein phenotype, and regulatory potential [44].

Paired multi-omic measurements reduce the uncertainty associated with computationally aligning modalities generated from different cells or samples and can help resolve relationships among chromatin accessibility, RNA expression, and protein phenotype. However, multi-omic workflows distribute limited molecular material and sequencing capacity across modalities, which can reduce the sensitivity of each modality relative to a dedicated single-modality assay. More importantly, the RNA component of most existing RNA–protein and RNA–chromatin assays remains based on poly(A) end counting. Single-cell total RNA sequencing should therefore not be viewed as a replacement for multi-omics, but as a potentially more complete intermediate layer connecting chromatin regulation, RNA processing, and protein function.

**Figure 1:**
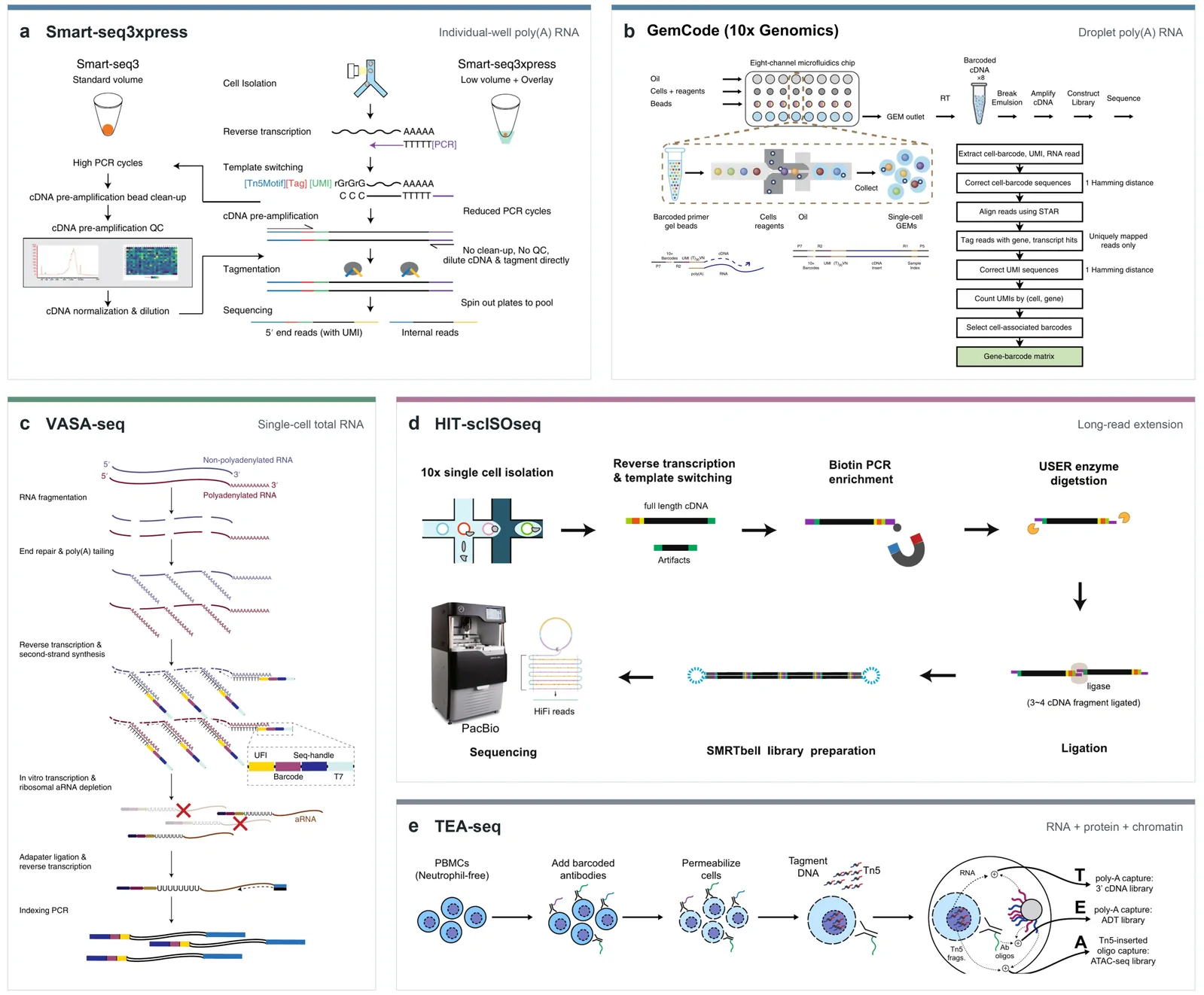
Workflow principles of representative single-cell profiling technologies. (**a**) The Smart-seq3 workflow is contrasted with its miniaturized Smart-seq3xpress implementation, which retains 5′ UMI-containing and internal gene-body reads. Adapted from ref. [24]. (**b**) GemCode (10x Genomics) combines droplet encapsulation with cell barcodes and UMIs for 3′ gene counting. Adapted from ref. [12]. (**c**) VASA-seq fragments and polyadenylates RNA to broaden the captured RNA repertoire. Adapted from ref. [18]. (**d**) HIT-scISOseq applies long-read sequencing to barcoded cDNA for isoform analysis. Adapted from ref. [40]. (**e**) TEA-seq jointly measures transcripts, surface epitopes, and chromatin accessibility. Adapted from ref. [44]. Panels (**a**–**e**) were adapted from the respective cited articles by cropping, resizing and rearranging source schematics; selected labels and graphical elements were redrawn for legibility. The source articles are available under the Creative Commons Attribution 4.0 International license (CC BY 4.0; https://creativecommons.org/licenses/by/4.0/).

Table 1 compares representative single-cell transcriptomic technologies by RNA capture spectrum, transcript coverage, reported cellular scale, and potential inputs for virtual-cell modeling. Figure 1 illustrates representative workflows from five technology classes; panel (a) also contrasts Smart-seq3 with its miniaturized Smart-seq3xpress implementation.

## 3. AI-Enabled Virtual-Cell Models Based on Single-Cell Omics

Single-cell omics data are inputs to, rather than complete instances of, virtual-cell models. A model trained on gene counts can learn useful associations and may predict unmeasured properties when supported by paired data or priors. However, the original assay does not directly observe those properties, and predictive success should be assessed independently on held-out biological contexts. Regulatory edges inferred from co-expression, motifs, or accessibility are candidate mechanisms until intervention or orthogonal evidence tests their direction and effect.

### 3.1. From Expression Matrices to Latent Cell States

A prerequisite for virtual cell construction is the conversion of high-dimensional expression matrices, which are affected by sparsity, sequencing depth, and batch effects, into stable and comparable representations of cellular state. Although conventional linear and nonlinear dimensionality-reduction methods can capture major sources of variation, they usually do not explicitly model the generative process underlying single-cell count data. Deep generative approaches address this limitation by treating observed transcript counts as measurements generated from an underlying biological state through sequencing and technical sampling processes.

Within this framework, scVI uses a variational autoencoder that takes raw UMI counts and experimental covariates as input. Its encoder infers a latent cellular state and library-size variables, whereas its decoder reconstructs gene counts using a negative-binomial or zero-inflated negative-binomial likelihood. Training is performed by maximizing the evidence lower bound, allowing the latent representation to retain biological variation while accounting for sequencing depth and batch-associated variation. The resulting state space supports data harmonization, clustering, differential-expression analysis, and uncertainty estimation, thereby providing a continuous coordinate system for virtual cell models [13]. Nevertheless, this latent representation captures statistical structure in the measured data and should not automatically be interpreted as a set of causal regulatory variables.

When only a subset of cells in a reference atlas has reliable annotations, representation learning can be coupled to cell-type labels. scANVI extends the scVI generative framework by introducing a discrete cell-type variable and jointly learning from labeled and unlabeled cells [45]. Query cells can consequently be mapped into the reference latent space and assigned posterior probabilities over cell types. This semi-supervised strategy is useful for atlas-scale label transfer, but its predictions depend on the taxonomy and state coverage of the reference dataset; genuinely novel states may be forced into previously defined categories.

RNA measurements alone may not adequately represent functional states at the cell surface. For datasets in which RNA and antibody-derived tags are measured in the same cells, totalVI uses a shared latent variable to model both modalities [46]. RNA counts are represented with a negative-binomial distribution, whereas protein measurements are described using a mixture model that separates background antibody signal from foreground protein signal. This formulation enables joint RNA–protein integration, background correction, differential analysis, and protein-level estimation in a common state space. However, the protein modality remains constrained by the predefined antibody panel and does not represent the complete proteome or protein-activity landscape.

Collectively, these generative models compress expression measurements into latent cell states that can be compared, interpolated and conditionally generated. They provide an essential starting point for virtual cell construction, but statistical continuity in a latent space does not necessarily imply continuity of the underlying biological process. Regulatory structure, temporal information and perturbational measurements are therefore required to give these representations mechanistic meaning and directionality.

### 3.2. Gene Regulatory Networks: From Association to Candidate Causal Edges

Cell-state representations describe how cells differ but do not directly explain which regulatory processes generate those differences. To predict the consequences of genetic interventions, a virtual cell must organize transcriptional covariation into candidate relationships among transcription factors, cis-regulatory elements and target genes.

One widely used strategy first identifies co-expression modules between transcription factors and potential targets and subsequently removes associations that lack supporting sequence evidence. SCENIC implements this strategy by using methods such as GENIE3 or GRNBoost to infer transcription factor–gene associations, followed by RcisTarget motif enrichment to refine co-expression modules into regulons [47]. AUCell is then used to quantify regulon activity in individual cells. Regulon-level activities are often more robust than the expression of individual transcription factors and can improve the identification of cell types and states. Nevertheless, co-expression and motif enrichment support only candidate regulatory relationships; they do not demonstrate that a transcription factor physically occupies and directly regulates a target locus in a particular cellular context.

The incorporation of chromatin accessibility enables regulatory inference to extend from genes to candidate enhancers. SCENIC+ first uses pycisTopic to identify accessible regulatory regions and state-associated accessibility programs from scATAC-seq data, applies pycisTarget to construct motif-supported transcription-factor cistromes, and then combines region-to-gene associations with transcription-factor evidence to infer enhancer-driven regulons, or eRegulons [48]. This procedure produces tripartite transcription factor–region–target gene relationships and can nominate the cis-regulatory elements through which a transcription factor may act. However, accessible chromatin and motif occurrence cannot substitute for direct measurements of transcription-factor occupancy, enhancer activity, or physical enhancer–promoter contact.

To support in silico intervention, a regulatory network must also estimate the direction and strength of regulation in specific cellular states. CellOracle begins with a base gene regulatory network derived from transcription-factor motifs and open-chromatin information and then applies regularized regression within cell clusters or local states to estimate context-specific regulatory coefficients [49]. Following simulated knockout or overexpression of a transcription factor, the predicted expression change is propagated through the network and projected onto a cell embedding or developmental trajectory. The resulting vector indicates how the perturbation might redirect cell-state transitions. These predictions remain dependent on the completeness of the base network, the regression assumptions, and the geometry of the low-dimensional projection, and should therefore be treated as experimentally testable mechanistic hypotheses.

Together, these approaches move virtual cell modeling from expression similarity toward explicit regulatory structure. A substantial gap nevertheless remains between a candidate regulatory edge and an interventionally established causal relationship. Perturb-seq, direct transcription-factor binding measurements, chromatin-conformation assays, and time-resolved experiments will be required to close this gap.

### 3.3. Dynamic Cell Models: Inferring Future Directions from Static Snapshots

Cells continuously change state, whereas most single-cell transcriptomic protocols require cell lysis and therefore provide destructive, cross-sectional measurements rather than longitudinal transcriptomic measurements of the same cell. Dynamic models must consequently reconstruct directionality from snapshots of different cells, RNA-processing states, or samples collected at multiple time points.

Splicing kinetics provide a source of local temporal information within snapshot data. RNA velocity compares the relative abundances of unspliced and spliced RNA and uses models of transcription, splicing and degradation to estimate the instantaneous rate of gene-expression change in each cell [15]. The resulting velocity vector points toward a putative successor state. Early implementations generally relied on steady-state or shared-kinetics assumptions and could therefore be biased during transient induction, non-steady-state regulation, or complex branching processes. scVelo models transient transcriptional induction and repression and estimates gene-specific transcription, splicing, and degradation rates, relaxing the steady-state approximation while retaining assumptions about the kinetic model and the stability of rate parameters within modeled phases. Its expectation-maximization procedure estimates gene-wise latent times, which are subsequently aligned to a shared cellular time scale for velocity analysis [50].

Metabolic labeling provides an additional means of distinguishing newly synthesized RNA from pre-existing RNA. Dynamo integrates metabolic-labeling and splicing information to estimate RNA velocities on a more nearly absolute scale and learns a continuous vector field in expression space [51]. This field can be used to identify fixed points, attractors and bifurcations, calculate local Jacobian matrices, analyze dynamical gene–gene responses and determine least-action paths between states. These analyses provide richer dynamical information than a conventional trajectory plot, although they rely on assumptions that the measured variables sufficiently specify the cellular state and that the local dynamics are continuous.

Local velocity vectors do not directly specify a cell’s eventual fate. CellRank converts local directional information into global fate probabilities by combining RNA velocity with a transcriptomic neighborhood graph to construct a directed Markov transition matrix [52]. Generalized Perron cluster analysis is then used to identify initial and terminal macrostates, after which absorption probabilities quantify each cell’s predicted tendency to reach alternative terminal states. These values represent model-derived fate propensities rather than experimentally observed lineage relationships and therefore require validation by lineage barcoding or time-resolved experiments.

When independent cells are sampled at multiple time points, population-level changes can instead be used to infer probabilistic ancestor–descendant relationships. Waddington-OT applies entropy-regularized optimal transport to identify a low-cost probabilistic coupling between cell distributions at adjacent time points [53]. Estimates of proliferation and death are incorporated to account for changes in cellular mass across states. The resulting transport maps can quantify how early populations contribute to later states, but they remain sensitive to sampling intervals, the distance metric used to represent cell states and the accuracy of growth-rate estimates.

RNA velocity, vector-field reconstruction, Markov fate modeling and optimal transport thus describe complementary aspects of cellular dynamics. They convert static atlases into directional state spaces, but they remain dynamical inferences from incomplete observations rather than direct records of the histories of individual cells.

### 3.4. Perturbation-Response Models: A Predictive Test for Virtual Cells

The ability to reconstruct an observed trajectory does not establish that a model can predict the outcome of an unseen intervention. A more stringent test of a virtual cell is whether it can generate a counterfactual expression state in response to genetic perturbation, drug treatment, dosage and cellular context. Technologies such as Perturb-seq provide paired measurements of perturbation identities and single-cell transcriptional responses, allowing this problem to be formulated as supervised or generative prediction.

Separating the intrinsic cellular background from the effect of an intervention is central to such prediction. The compositional perturbation autoencoder (CPA) uses an encoder to extract a basal cell state from the observed expression profile and applies adversarial training to reduce perturbation and covariate information remaining in this representation [54]. It then learns separate embeddings for chemical or genetic perturbations, dose, and covariates such as cell type. These components are compositionally added to the basal state, and a decoder generates the predicted post-treatment expression profile. This architecture can generalize to new combinations of represented perturbations, doses, and cellular contexts, but it cannot infer a completely unseen perturbation solely from its name when no molecular representation of that intervention is available.

Combinatorial genetic perturbations additionally require a representation of functional relationships among genes. GEARS uses a graph neural network operating on a gene co-expression graph to learn gene-state representations and a second graph derived from Gene Ontology relationships to propagate functional information from perturbed genes [55]. For multigene interventions, the perturbation representations are combined and passed through cross-gene propagation layers to predict gene-specific expression changes. These graph structures provide inductive priors that support generalization to unseen combinations, although performance depends on graph quality, coverage of single-gene perturbations, and the similarity between training and target cellular contexts.

Systematic evaluations have shown that high predictive correlations may partly reflect a response shared across many perturbations rather than accurate reconstruction of perturbation-specific effects. The Systema framework demonstrated that mean-response and linear baselines can match or outperform more complex models in several datasets [56]. Evaluation should therefore distinguish systematic shifts from perturbation-specific variation and employ held-out tests defined by perturbation identity, cell context or gene combination.

Perturbation modeling is consequently a crucial step in moving virtual cells from description to prediction. Yet counterfactual capabilities of current models generally remain local to the training distribution. A reliable virtual cell should report not only a predicted response but also its uncertainty, domain of applicability, and experimentally falsifiable consequences.

### 3.5. Single-Cell Foundation Models: Large-Scale Pretraining and Its Limits

As public single-cell datasets have expanded to tens or hundreds of millions of cells, model development has shifted from training on individual datasets toward pretraining transferable representations on large corpora. The underlying premise is that recurrent patterns of gene covariation across tissues, diseases and experimental conditions may encode general biological regularities that can be transferred to cell annotation, perturbation prediction and regulatory-network analysis.

Inspired by masked language modeling, Geneformer ranks genes within each cell according to their expression and corpus-wide abundance, represents highly ranked genes as an ordered token sequence, and uses a Bidirectional Encoder Representations from Transformers (BERT) architecture to predict masked gene tokens [16]. The pretrained representation can be fine-tuned for cell classification, disease-state identification and in silico gene deletion. However, rank-based encoding deemphasizes absolute abundance, zero expression, dosage-dependent variation and transcript isoform information.

An alternative strategy represents both gene identity and expression magnitude. scGPT treats genes as tokens, encodes discretized expression values as associated numerical embeddings and adds condition or modality information before applying a Transformer to masked expression-value prediction [17]. Task-specific fine-tuning enables the same pretrained model to support cell annotation, batch integration, multi-omics integration, gene-network inference and perturbation-response prediction. This unified interface is highly extensible, although discretization loses some quantitative resolution and attention weights should not be interpreted directly as causal regulatory relationships.

Broad gene coverage and computational scalability become important when models approach the complete human gene vocabulary. scFoundation uses an asymmetric encoder–decoder architecture and performs read-depth-aware masked-expression reconstruction on more than 50 million cells [57]. Its encoder focuses primarily on observed non-zero genes, whereas the decoder reconstructs the complete gene space, reducing the cost of applying self-attention to approximately 20,000 genes simultaneously. Although this design supports a broad feature vocabulary, platform-specific biases and technical dropout in the pretraining corpus may also be absorbed by the model.

Cellular states are additionally influenced by tissue organization and neighboring cells, making independent-cell representations incomplete in spatial contexts. CellPLM treats cells as tokens and tissue sections or local spatial neighborhoods as multicellular “sentences” [58]. Individual expression profiles are first encoded at the cell level, after which an intercellular Transformer learns contextual relationships and reconstructs masked cell profiles from neighboring cells. This design is well suited to spatial transcriptomic and tissue-level modeling, but its output depends on neighborhood definitions, spatial resolution, and the representation of tissue architectures in the training data.

Cross-species generalization requires representations that extend beyond a fixed human gene vocabulary. UCE derives evolutionary representations of protein-coding genes from a protein language model, samples non-zero genes according to their expression levels, organizes them using chromosome and genomic-position information and applies a Transformer to learn universal cell embeddings [59]. This design enables zero-shot application across multiple species, but it is currently centered on protein-coding genes, and expression-weighted sampling sacrifices some precise quantitative information.

A further increase in both corpus and parameter scale is represented by CellFM. The model was pretrained on approximately 100 million human cells with roughly 800 million parameters [60]. Scalar expression projections are combined with gene-identity embeddings, and an ERetNet architecture with near-linear computational complexity performs masked-expression reconstruction; a cell-level token provides the global representation. Parameter-efficient adaptation to downstream tasks is supported through low-rank adaptation. Importantly, most of the pretraining corpus consists of 10x Genomics 3′ expression data, meaning that greater model scale does not automatically overcome the informational boundary of poly(A) gene-count matrices.

Neither parameter count nor the number of pretraining cells guarantees superior biological generalization. Independent zero-shot evaluations have found that Geneformer and scGPT do not consistently outperform simpler approaches, including highly variable gene representations, Harmony and scVI, in tasks such as clustering and batch integration [61]. Evaluations should therefore distinguish pretraining reconstruction performance, task-specific performance after fine-tuning and genuine zero-shot transfer without target-dataset labels.

Single-cell foundation models provide a scalable framework for AI-based analysis, but they inherit the molecular coverage, dynamic range, missingness and platform biases of their training measurements. Because most current corpora consist of gene-level count matrices generated by high-throughput poly(A)-based scRNA-seq, these models primarily learn statistical regularities within an assay-defined gene-expression space rather than the complete molecular state of the cell. Total-RNA and transcript-resolved assays may expand this space by detecting additional non-polyadenylated RNAs, transcript isoforms, RNA-processing intermediates, and repeat-derived transcripts, subject to assay-specific capture and readout limitations. The predictive value of these added features should not be inferred from improved reconstruction alone. It should be tested through matched comparisons that hold model architecture, training design and sequencing investment constant while assessing generalization to unseen samples, platforms and perturbations, together with uncertainty calibration and mechanistic interpretability.

**Figure 2:**
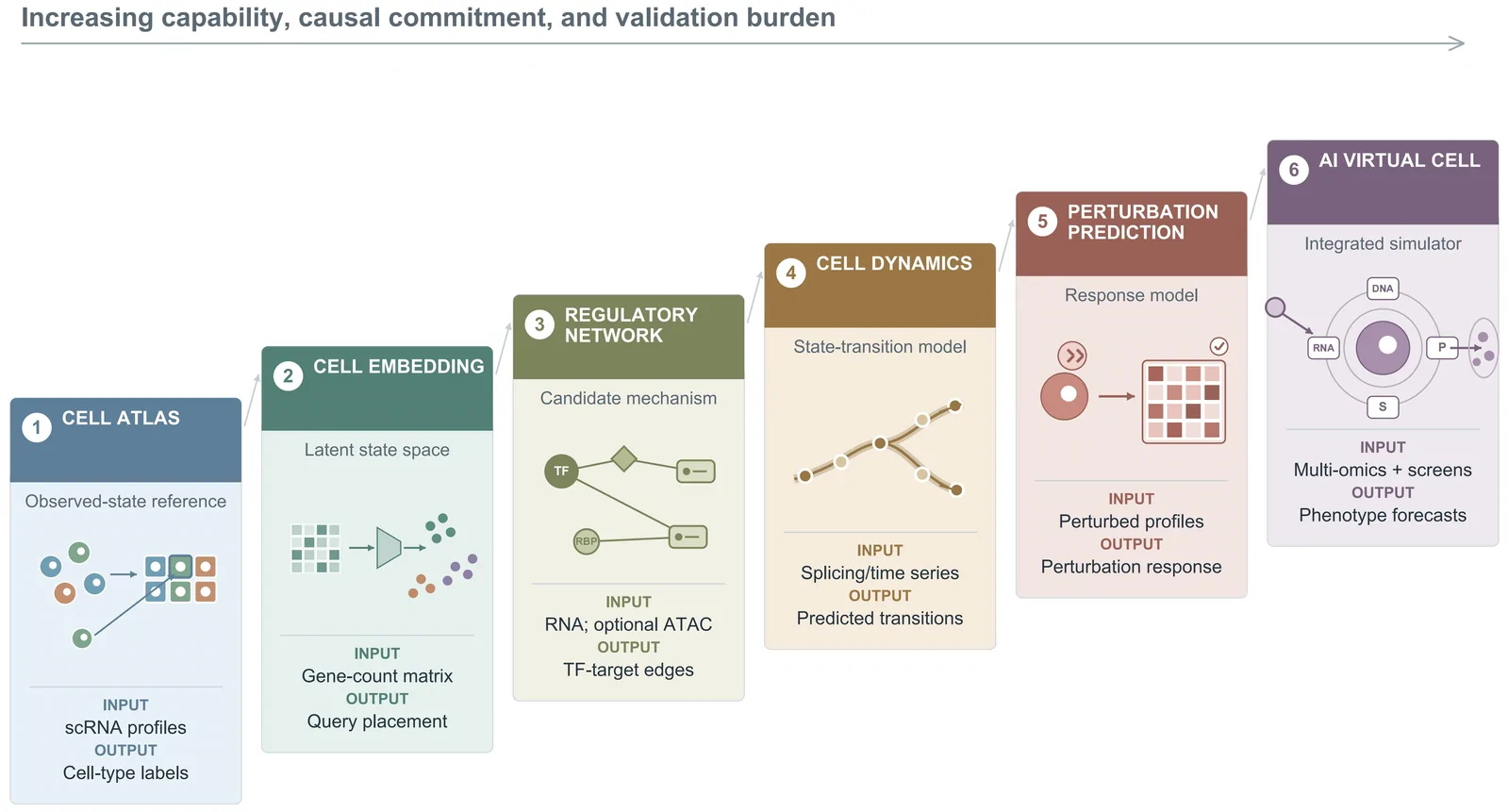
Capability ladder for virtual-cell modeling. The six stages progress from a cell atlas and cell embedding to regulatory networks, cell dynamics, perturbation prediction, and an AI virtual cell. Each stage shows a representative measurement or training input and a testable output; the inputs are illustrative rather than universal requirements. Regulatory edges and predicted cell transitions remain hypotheses until evaluated using appropriate perturbations, time-course data, or independent functional measurements. Progress along the ladder entails increasing causal commitment and validation demands, but does not imply that any existing model fulfills all six capabilities.

The major functional layers of current virtual-cell models, together with their representative methods, typical inputs and principal limitations, are summarized in Table 2. The evidence underlying these model categories—including the assays and features actually used as inputs, whether single-cell total-RNA data were included, benchmark tasks, reported evaluation settings, uncertainty assessments and principal limitations—is summarized in Table 3. The relationships among these modeling layers and their progressively more demanding predictive aims, causal commitments, and validation requirements are illustrated in Figure 2.

## 4. How Single-Cell Whole-Transcriptome Detection Could Inform Predictive Virtual-Cell Models

Expanding RNA capture changes what a virtual-cell model can observe, but the value of an additional signal depends on what can be demonstrated with it. In this section, we distinguish molecular features detected experimentally from computational gains established through matched comparisons and from modeling applications that remain prospective. Detecting an RNA species shows that it is observable under specified assay conditions; it does not, by itself, establish improved prediction in an independent biological context or identify a causal regulator. Such claims require evidence matched to the question being asked.

Accordingly, we describe a signal as measured when it is reproducibly detected, associated when it shows a statistical relationship with another variable, and predictive when a model using it performs on held-out biological contexts. A proposed relationship is mechanistically interpretable when it offers a plausible, testable explanation, but causally validated only when supported by an appropriately designed intervention and independent functional evidence. Here, identifiability means that the available observations, under stated assumptions, can distinguish a proposed biological quantity or mechanism from plausible alternatives. A model may predict an unmeasured feature from correlated or paired data, but that feature is not thereby directly measured or causally identified. These evidentiary categories are not mandatory sequential stages. Rather, they clarify the level of evidence behind each claim and help separate the broader RNA observability offered by these assays from demonstrated improvements in virtual-cell modeling.

### 4.1. From Gene Abundance to Transcript-Resolved State Variables

Most current virtual-cell models represent a cell as a vector comprising the abundances of thousands of genes. This representation is effective for cell-type identification and broad state comparisons, but generally collapses multiple transcripts from the same gene into a single value. Alternative splicing, transcription initiation and transcript degradation can alter coding potential, stability, localization and translational efficiency without substantially changing total gene abundance. Consequently, two cells with similar gene-expression matrices may still occupy different RNA-processing states and possess different functional potentials.

VASA-seq demonstrated that relatively uniform gene-body coverage and capture of non-polyadenylated RNAs increase the observability of splicing events and non-coding RNAs [18]. Single-cell long-read sequencing further revealed transcript isoform programs with cell-type-, brain-region- and developmental-stage-specific patterns that cannot be fully explained by gene-level expression [62]. TotalX showed that total-RNA capture can be adapted to standard droplet microfluidics while recovering protein-coding transcripts, lncRNAs and subsets of small RNAs [35]. Together, these technologies could expand the state space of virtual cells from a gene-by-abundance matrix to a hierarchical representation containing genes, isoforms, splice junctions and RNA biotypes. Short-read total-RNA sequencing and long-read isoform sequencing nevertheless provide complementary information; broad non-poly(A) coverage should not be equated with complete isoform resolution. These additional RNA and transcript-level features are therefore candidate state variables; whether they improve virtual-cell predictions beyond matched gene-count baselines remains a task-specific empirical question.

### 4.2. Incorporating Non-Coding and Post-Transcriptional Regulatory Features

Transcriptional activity in the human genome extends far beyond canonical protein-coding genes, with substantial signals arising from lncRNAs, antisense RNAs, enhancer RNAs and incompletely annotated regions [63,64]. RNA-binding proteins regulate splicing, polyadenylation, nuclear export, localization, translation and degradation, forming a regulatory layer between transcription and cellular function [65]. Circular RNAs, miRNAs and other small RNAs can further reshape cellular states through competitive binding, target-RNA degradation or translational repression [66,67].

Conventional 3′ poly(A)-based gene-count data can reveal some RNA-processing signals, particularly 3′-end usage, but provide limited access to many non-polyadenylated RNAs and transcript-wide processing events. Depending on assay design, broader RNA capture and complementary measurements may detect additional candidate regulatory RNAs and RNA-processing products alongside potential target transcripts. Joint single-cell miRNA–mRNA measurements have revealed cell-to-cell anticorrelations between miRNA abundance and predicted target expression [67]. Analysis of single-cell 3′-end data has revealed cell-type- and developmental-stage-dependent alternative polyadenylation (APA), reflected in changes in 3′ untranslated region (3′-UTR) length [68], whereas locus-resolved analyses of transposable elements can distinguish expression from individual genomic copies instead of collapsing signals at the family level [69]. These readouts can supply candidate non-coding RNA and RNA-processing features for multilayer regulatory models. Assigning regulatory roles or edges to RNA-binding proteins additionally requires orthogonal binding or perturbation evidence. Co-detection alone does not establish the direction or causal effect of a proposed regulatory relationship.

### 4.3. Providing More Direct Observables for Dynamical Modeling

Conventional scRNA-seq generally requires cell lysis and therefore yields terminal, cross-sectional molecular profiles rather than longitudinal measurements of the same cell. RNA velocity uses the relative abundance of unspliced and spliced RNA to estimate local directions of state change from a population of static snapshots [15], while scVelo uses a dynamical model that relaxes the steady-state approximation while retaining assumptions about kinetic form and rate parameters [50]. The broader gene-body coverage of VASA-seq increases unspliced-RNA recovery and may improve velocity inference in particular developmental systems [18]. However, additional unspliced reads do not directly measure RNA synthesis or degradation rates, whose estimation still depends on kinetic assumptions or complementary labeling and time-course measurements.

Steady-state RNA abundance is nevertheless determined jointly by synthesis, processing and degradation, and additional unspliced reads alone cannot uniquely identify these kinetic parameters. Metabolic labeling distinguishes newly synthesized from pre-existing RNA and provides temporal constraints on estimates of RNA synthesis and turnover; rate estimation still depends on labeling design and kinetic assumptions. scSLAM-seq, NASC-seq, sci-fate and scEU-seq have demonstrated the feasibility of measuring nascent transcription and RNA renewal at single-cell resolution [70,71,72,73], while models such as Dynamo use metabolically labeled data to estimate vector fields and state-transition dynamics [51]. Combining broader RNA capture with temporal labeling may therefore allow virtual cells to model RNA production, processing and clearance instead of fitting trajectories solely from mature mRNA snapshots. These measurements still predominantly sample different cells, however, and should not be regarded as complete substitutes for longitudinal live-cell tracking.

### 4.4. Expanding the Feature Vocabulary and Learning Objectives of Foundation Models

Current single-cell foundation models generally treat genes as their basic feature units and encode expression through rankings, discretized values or learned embeddings. Geneformer, scGPT, scFoundation and CellFM have shown that large single-cell corpora can support transferable cell representations, gene-network inference and perturbation prediction [16,17,57,60]. When the training corpus consists primarily of poly(A) gene-count matrices, however, the learned representation remains a restricted projection of the cellular RNA state.

RNA features from total-RNA and transcript-resolved assays could expand the model vocabulary to include transcript isoforms, splice junctions, poly(A) sites, non-coding RNA classes and locus-resolved transposable-element-derived transcript features. A single-cell long-read study showed that isoform-level features reveal cell-type- and developmental-stage-specific patterns not fully captured by gene-level abundance [62]. Whether the other proposed RNA features improve model performance remains to be tested in matched comparisons. Pretraining objectives could accordingly extend beyond gene-expression reconstruction to include isoform-usage prediction, masked splice-junction modeling, nascent-to-mature RNA mapping, miRNA–target consistency and cross-biotype representation learning. A larger vocabulary does not automatically yield a better model: low abundance, multimapping, incomplete annotation and platform-specific missingness may instead introduce noise. Transcript- and event-level tokens remain proposed extensions of foundation-model vocabularies; whether they improve cross-dataset generalization, dynamical prediction or perturbation modeling must be tested through feature ablations and held-out evaluations, rather than inferred from vocabulary size.

### 4.5. Redefining Perturbation Responses and Causal Validation

Perturb-seq combines genetic perturbations with single-cell transcriptional readouts, allowing models to learn mappings from interventions to cellular-state changes [14]. Standard gene-count outputs from poly(A)-based assays collapse many RNA-processing responses, although the underlying 3′ reads can sometimes support inference of poly(A)-site usage. A 3′ scRNA-seq screen of 42 cleavage and polyadenylation regulators, termed CPA-Perturb-seq, identified modular changes in poly(A)-site usage [74].

Adding total-RNA or transcript-resolved readouts to single-cell perturbation experiments may reveal intermediate RNA responses missed by gene-level counts, but whether these signals improve counterfactual prediction must be demonstrated using matched training data and held-out evaluations. Scalable total-RNA approaches such as TotalX provide a technical basis for measuring non-coding and small RNAs across larger cell populations [35], although their compatibility with complex perturbation libraries, sequencing cost and statistical power require systematic evaluation. Such models should be assessed separately at the levels of gene abundance, isoform usage, RNA biotype and functional phenotype instead of compressing all responses into a single expression-correlation metric.

### 4.6. Connecting Subcellular Organization, Tissue Space and the Microenvironment

An RNA state is defined not only by molecular abundance but also by localization. Nuclear retention, nuclear export, mitochondrial or endoplasmic-reticulum localization and local translation can confer different functions on otherwise identical RNA molecules. scRICA-seq pairs isoform profiles with chromatin accessibility in individual cells [75], providing regulatory context rather than subcellular RNA coordinates. In a separate proximity-labeling assay, APEX-seq maps RNA localization across subcellular compartments [76].

At the tissue scale, spatial total-RNA sequencing based on in situ polyadenylation expanded the detection of non-polyadenylated, unspliced and viral RNAs and related their distributions to regions of injury and infection [77]. Single-cell total-RNA data may therefore characterize intracellular RNA-regulatory states, whereas spatial total-RNA and imaging measurements can supply tissue position, neighboring-cell and microenvironmental constraints. Total-RNA capture alone does not establish subcellular position; localization and tissue-niche effects require spatial, imaging, fractionation, or appropriately paired measurements. Integrating these readouts with transcriptomic profiles may enable virtual-cell models to represent subcellular organization and tissue context.

**Figure 3:**
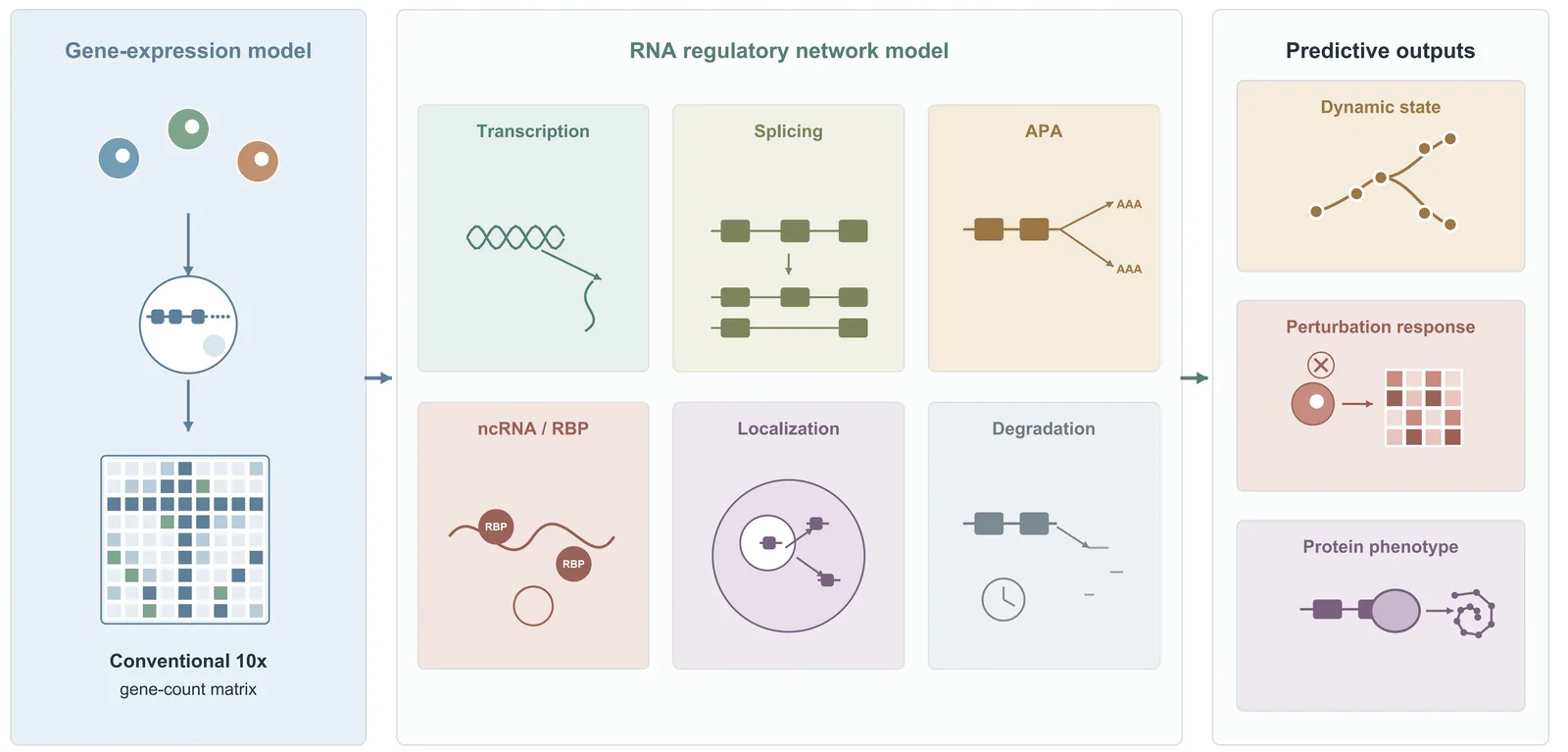
From a gene-expression model to an RNA regulatory-network model. Conventional 10x Genomics poly(A)-based workflows typically yield gene-level count matrices. The central panel depicts regulatory processes that a virtual-cell model may represent: transcription, splicing, alternative polyadenylation (APA), non-coding RNA and RNA-binding protein (ncRNA/RBP) regulation, localization, and degradation. Broader RNA capture can reveal additional RNA biotypes and processing intermediates, but no single assay directly measures all six processes. Transcript-structure analysis depends on library design and molecular coverage; direct RNA–protein interactions, subcellular localization, and RNA synthesis or degradation rates require complementary binding, spatial or imaging, and temporal measurements, respectively. Dynamic states, perturbation responses, and protein phenotypes are potential model outputs, not benefits established by broader RNA capture alone. Predictive gains require comparison with matched gene-count baselines, while proposed causal relationships require perturbation and independent functional validation.

The resulting expansion from a conventional gene-expression model to an RNA regulatory-network model is schematically illustrated in Figure 3.

### 4.7. Analytical Performance, Standardization and Evidentiary Boundaries

The analytical performance of broader RNA capture must be weighed against its additional molecular coverage. Without effective depletion, rRNA and other abundant species may consume a substantial share of sequencing reads, reducing informative non-rRNA depth at a fixed budget. Recovery can also vary with RNA length, guanine–cytosine (GC) content, secondary structure, and ligation or tailing efficiency. Repetitive and transposable-element-derived reads may map ambiguously, while incomplete annotations can cause genuine non-coding signals to be missed [35,69]. Assay comparisons should therefore distinguish cell recovery—the fraction of input cells yielding usable profiles—from estimated RNA-molecule recovery or capture efficiency. They should also report library complexity, the informative non-rRNA read fraction, RNA-biotype-specific detection, background signal, and sequencing saturation [20,78,79]. Costs should be stated per usable cell and, where feasible, per reproducibly detected task-relevant feature. At a fixed total budget, profiling more cells may leave insufficient depth for low-abundance RNAs, whereas sequencing fewer cells more deeply may limit the diversity of states sampled. These trade-offs depend on tissue, sample preparation, whether cells or nuclei are profiled, and the RNA classes of interest.

To determine whether the additional signals benefit virtual-cell modeling, poly(A)-based and broader-RNA assays should be applied to parallel aliquots of matched biological samples, with cell composition assessed and batches randomized. Because library preparation generally consumes the sampled cells, this design does not profile the same physical cell twice. Two complementary comparisons are useful: equal reads per profiled cell and equal total experimental budget. Cell recovery and informative non-rRNA depth should remain explicit outcomes in both, rather than being normalized away. Where feasible, preprocessing, feature definitions, model architecture, optimization budget, and training–test partitions should be harmonized, with unavoidable assay-specific differences documented. Gene-only models trained on each assay, together with simple baselines, should be compared with expanded-feature models. This helps separate gains due to added RNA information from those due to platform effects, greater depth, or model capacity.

Validation should reflect the intended use of the model. Holding out donors, tissues, platforms, or perturbations tests generalization more directly than relying only on random splits of closely related cells. Depending on the task and available ground truth, outcomes should include reproducible detection of added features, prediction of state transitions or perturbation responses, calibration of predictive uncertainty, and independent tests of proposed mechanisms or phenotypes. Broader RNA capture should be considered beneficial for a specified virtual-cell task only when it produces reproducible gains over a matched gene-count baseline under clearly stated measurement and budget constraints.

**Figure 4:**
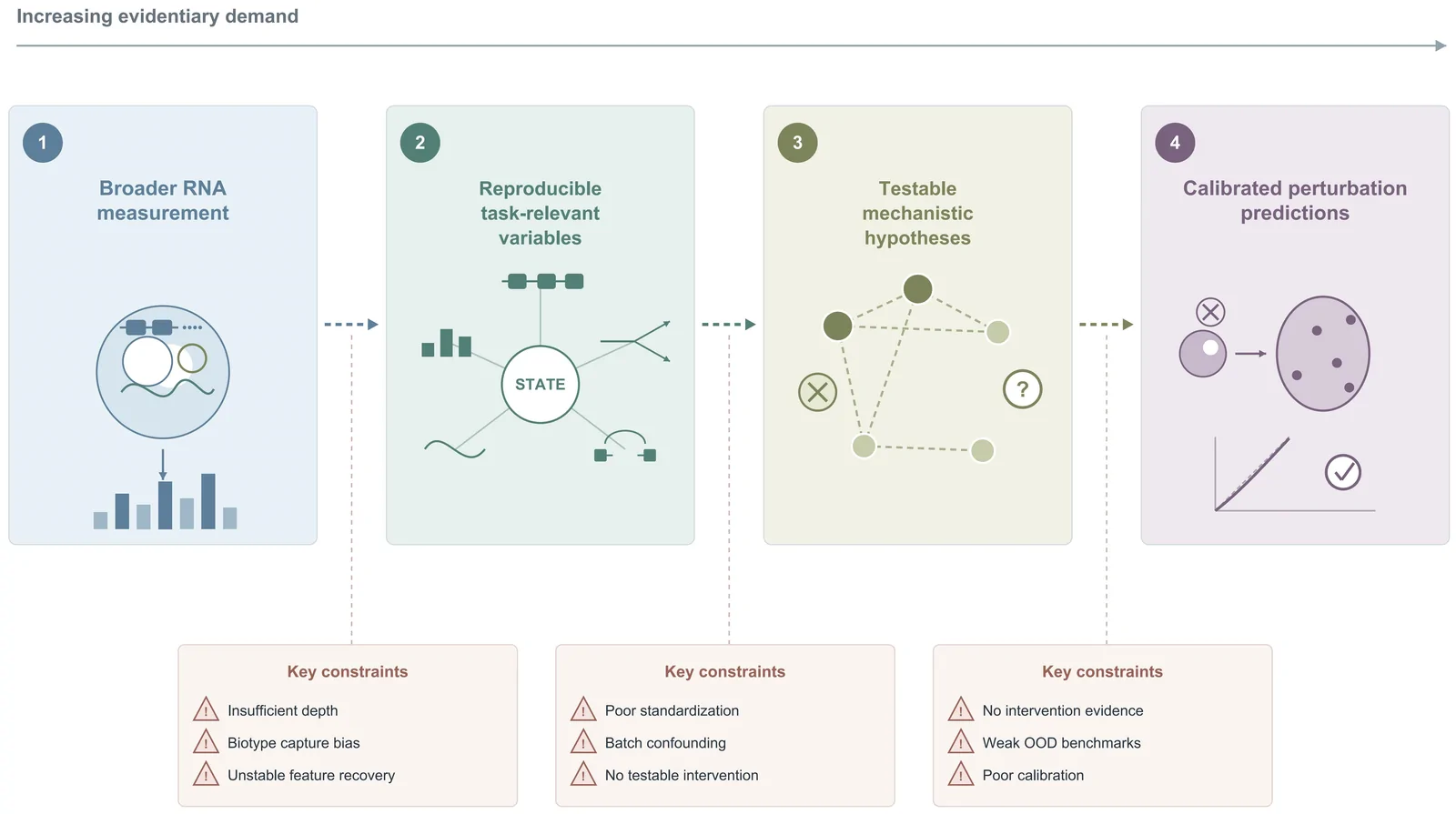
Conditional evidence pathway from broader RNA measurement to calibrated perturbation prediction. The four stages distinguish broader measurement, reproducibly recovered task-relevant variables, testable mechanistic hypotheses, and calibrated predictions for unseen perturbations. Dashed arrows indicate claims that require testing, not automatic causal steps. Limited informative depth, RNA-biotype-specific capture bias, or unstable feature recovery can prevent reliable variables; poor standardization and confounding can distort mechanistic hypotheses; and absent intervention evidence, out-of-distribution (OOD) evaluation, or poor calibration can undermine predictive claims. Statistical reconstruction of an unmeasured RNA feature is not direct molecular measurement. Causal identification requires explicit assumptions and appropriately designed interventions, supported by orthogonal evidence. Gains in virtual-cell prediction must be demonstrated against matched gene-count baselines under stated measurement and budget constraints.

Table 4 distinguishes observed RNA-related signals from their proposed modeling uses, required validation, and current evidence status. Figure 4 illustrates the conditional progression from broader RNA measurement to reproducible task-relevant variables, testable mechanistic hypotheses, and calibrated perturbation predictions, highlighting constraints at each transition. Table 5 outlines a proposed matched benchmark for testing whether additional RNA features improve virtual-cell predictions beyond gene-count baselines under defined sequencing and budget constraints.

## 5. Future Perspectives

Future progress will require the co-design of molecular measurement and AI modeling rather than the indiscriminate maximization of assay dimensionality. Broader RNA measurements may contribute additional state variables, but their value for virtual-cell prediction must be demonstrated in task-specific comparisons with matched gene-count baselines. Chromatin, protein, spatial, temporal, and perturbation data can provide complementary constraints when relevant to the task. A practical strategy is to construct hierarchical datasets aligned with specific predictive tasks: high-throughput assays provide breadth across cells, individuals and conditions; total-RNA and long-read assays provide complementary regulatory detail in representative states; and matched samples or partially co-assayed bridge cells connect RNA with additional modalities. Within this framework, integration with multi-omics, AI foundation models, spatial technologies and longitudinal patient measurements defines four major directions for measurement-informed virtual cells.

### 5.1. Integrating Single-Cell Whole-Transcriptome Detection with Multi-Omics

No individual omics layer can independently define the complete cellular state. Chromatin accessibility and epigenetic modifications describe the regulatory potential of genomic elements, RNA reflects the combined output of transcriptional and post-transcriptional regulation, and proteins provide a readout more closely connected to signaling and effector functions. Single-cell multi-omics technologies have already enabled joint measurements of RNA, chromatin accessibility, and proteins [80]. CITE-seq links poly(A) RNA expression with surface-protein abundance [41]; sci-CAR and SHARE-seq jointly profile chromatin accessibility and RNA expression [42,43]; and TEA-seq and DOGMA-seq extend these measurements to trimodal profiles of RNA, chromatin accessibility and surface proteins [44,81]. These paired measurements can distinguish cells with different chromatin-accessibility, RNA-expression, and surface-protein profiles; causal relationships among these layers require perturbation or time-resolved evidence.

The RNA component of many current multi-omic assays is a poly(A)-based gene-count readout, with limited access to many non-polyadenylated RNAs and transcript-wide processing events. Replacing this component with a broader total-RNA readout is not a simple exchange of library chemistries. Permeabilization for ATAC profiling may cause cytoplasmic RNA loss; fixation and nuclear extraction can alter the recovery of different RNA classes; and rRNA depletion, in vitro tailing and small-RNA retention may compete with antibody-tag or chromatin-library preparation. Dedicated co-assays will therefore be required, together with modality-specific reporting of recovery efficiency, cross-contamination, cell loss and allocation of sequencing capacity.

For large studies, a more realistic design may combine a paired core with a mosaic extension. A limited number of bridge cells could be jointly profiled for a broader RNA repertoire, chromatin accessibility, and proteins, whereas a much larger population could be measured using less expensive single- or bimodal assays. MultiVI illustrates mosaic integration by combining paired gene-expression and chromatin-accessibility profiles with single-modality datasets; the original study also demonstrated an extension incorporating antibody-derived surface-protein measurements [82]. These experiments did not evaluate single-cell total-RNA inputs, so applying this strategy to broader RNA measurements remains to be tested. Imputed modalities should be treated as model predictions rather than direct observations and evaluated using paired data excluded from training, particularly for states poorly represented in the training set.

Ultimately, this integration should support a hierarchical model extending from chromatin opening and transcription initiation through nascent-RNA production, RNA processing and isoform selection to protein accumulation. Temporal and perturbational constraints will be essential for converting these cross-modal associations into directional regulatory paths.

### 5.2. Integrating Broader RNA Features with AI-Based Virtual-Cell Models

Current single-cell foundation models generally treat genes as tokens or basic feature units and are pretrained predominantly on large poly(A)-based gene-count matrices [16,17,57,60]. One possible design for future foundation models incorporating broader RNA features is a hierarchical feature system. Genes may provide a relatively stable upper-level vocabulary, whereas transcript isoforms, splice junctions, poly(A) sites, non-coding RNAs and locus-resolved transposable-element-derived transcript features provide more granular RNA features. Chromatin regions, proteins and spatial neighborhoods can then be represented as separate modalities linked to the RNA state. Such a hierarchy would preserve relationships among genes, transcripts and regulatory events without forcing every molecular observation into gene-level abundance.

Computational analysis of biosensing data can be organized into three connected levels. At the signal-processing level, computational methods perform barcode and UMI correction, read assignment, isoform quantification, background removal, and quality assessment. At the representation level, modality-specific observation models integrate heterogeneous molecular measurements while preserving assay-specific uncertainty. At the predictive level, models infer regulatory relationships, cellular dynamics and responses to genetic or pharmacological perturbations. These levels should be evaluated separately: improved denoising or data reconstruction does not necessarily demonstrate improved biological prediction. Conversely, a predictive model should not be credited with recovering a molecular mechanism when that mechanism is not identifiable from the measured signals.

A potential architecture would use modality-specific encoders with appropriate observation models, a shared latent representation of cellular state, and modality-specific decoders. The model should support not only annotation and expression reconstruction but also prediction of nascent transcription from chromatin, mature RNA and isoform composition from nascent RNA, protein changes from RNA states, and future cellular states from perturbation conditions. GET has demonstrated that a foundation model combining genomic sequence and chromatin accessibility can learn transcriptional regulatory rules across cell types and predict gene expression [83]. Incorporating these additional RNA features could extend predicted outputs beyond gene abundance to splicing and polyadenylation; predictions of RNA stability would additionally require suitable temporal or kinetic training data and independent validation.

Training corpora for such models should not consist solely of increasing numbers of unperturbed cells. A hierarchical corpus could use high-throughput poly(A)-based scRNA-seq for broad tissue and cell-type coverage, total-RNA sequencing for non-coding and RNA-processing information in representative states, long-read sequencing for isoform structures, and temporal, metabolic-labeling, and Perturb-seq data for transition directionality. Training objectives could combine within-modality masked reconstruction, cross-modal prediction, temporal-order discrimination, and generation of perturbation responses.

Evaluation practices must also change. Randomly splitting cells into training and test sets often places highly similar biological samples in both subsets and can substantially overestimate generalization. More stringent evaluations should leave out entire donors, tissues, diseases, experimental platforms or perturbation classes and separately assess cross-platform transfer, zero-shot prediction, and uncertainty calibration. Existing assessments have shown that single-cell foundation models do not consistently outperform smaller task-specific models on every application [61]. Their value should therefore be determined by predictive performance on unseen states and interventions, rather than parameter count or the number of cells used for pretraining.

### 5.3. Integrating Single-Cell Whole-Transcriptome Detection with Spatial Transcriptomics

Tissue dissociation removes anatomical positions, neighborhood relationships, and microenvironmental exposures, encouraging virtual cells to be represented as isolated objects outside their tissue context. In reality, cellular states are jointly influenced by local ligands, extracellular matrix, vasculature, immune infiltration, oxygen and nutrient gradients, and the spatial distribution of pathogens. A more precise future concept is therefore not simply a “spatial virtual cell”, but a spatially conditioned virtual cell whose behavior depends on tissue location and neighborhood state.

Spatial total-RNA sequencing has demonstrated that in situ polyadenylation can increase the detection of non-polyadenylated, unspliced and viral RNAs in tissue sections and relate these signals to regions of injury or infection [77]. Spatial ATAC–RNA-seq and spatial CUT&Tag–RNA-seq further showed that chromatin accessibility or histone modifications can be co-mapped with RNA expression in the same tissue section, enabling analysis of epigenetic priming and transcriptional output within tissue architecture [84]. High-density approaches such as Stereo-seq have also demonstrated the feasibility of constructing large-scale developmental atlases at or near cellular resolution [85].

A spatial virtual-cell model should represent at least three scales: the intracellular RNA state characterized by broader profiling, the local niche formed by neighboring cells and tissue structures, and tissue-scale fields such as drug, cytokine, oxygen or metabolite gradients. Cells can be represented as nodes in a graph, physical contact, proximity or ligand–receptor relationships as edges, and continuous tissue gradients as spatial fields. The resulting model could predict how the same cell changes across different neighborhoods or generate counterfactual outcomes after removing a neighboring population or blocking a local signal.

Spatial colocalization, however, does not establish intercellular communication. Spatial resolution, segmentation errors, RNA diffusion and section orientation may create apparent associations, while co-expression of a ligand and receptor does not demonstrate pathway activation. Spatially conditioned virtual cells will therefore require validation through spatial perturbations, in situ imaging, tissue culture or organoid experiments. A convincing model should predict the direction and magnitude of RNA-state changes after altering neighborhood composition or local signaling, rather than merely reconstructing an observed tissue map.

### 5.4. From Virtual-Cell Models to Entity-Specific Digital Twins

Longitudinal single-cell and spatial atlases are beginning to describe changes in cellular states during tumor progression and treatment. The Human Tumor Atlas Network provides an important framework for connecting multiparametric and spatial measurements with clinical context [86]. Such resources can supply reference states and population-level priors, but they do not themselves constitute digital twins. A longer-term extension of virtual-cell modeling is an entity-specific digital twin: a computational model repeatedly aligned with measurements from a defined biological entity and used to generate testable predictions [87,88,89,90].

The entity may be an individual cell or cell population, tissue, animal, or patient. At each scale, the tracked unit and the way new observations update the model must be specified. Because conventional single-cell sequencing consumes the measured cell, repeatedly tracking the same physical cell requires complementary, non-destructive measurements; sequencing-based models will more often follow changes in cell-state or clonal distributions. For patient-level applications, atlases and virtual-cell models may provide priors, whereas longitudinal clinical and molecular observations are needed for individual calibration. A single baseline transcriptomic profile, however detailed, is not a digital twin.

One possible patient-oriented workflow would combine standardized baseline poly(A)-based profiling for cellular breadth with selected total-RNA, long-read, spatial, and protein measurements when they provide task-relevant information. Analytical quality control would first establish whether the required states and RNA features are detected reproducibly. Follow-up imaging, circulating biomarkers, repeat sampling when clinically appropriate, and perturbation experiments using patient-derived material could then update and test the model. Where adequately validated, its outputs should include predictive uncertainty, measurement-quality indicators, and explicit conditions under which a prediction should not be used.

Validation should proceed from retrospective longitudinal cohorts to prospective studies that fix the model before predicting future outcomes; model-guided interventions require a further stage of clinical evaluation. Patient-level applications must also address privacy, population bias, platform drift, interoperability, principles for findable, accessible, interoperable, and reusable data [91], and regulatory oversight. At present, broader RNA profiling may refine the initial molecular state and prioritize testable response hypotheses. Reliable model-guided treatment selection remains a prospective goal.

## 6. Conclusions

Cell atlases map observed states, whereas virtual-cell models seek to predict how those states change. High-throughput poly(A)-based single-cell RNA sequencing has provided the cellular breadth and gene-count data underlying many current models, but captures only part of RNA regulation. Broader single-cell total-RNA methods reveal additional non-coding and unspliced RNAs and processing signals. Long-read sequencing, metabolic labeling, spatial profiling, and multi-omics provide complementary structural, temporal, positional, and non-RNA information. No single assay supplies all these dimensions, and broader RNA capture alone does not establish localization, kinetic rates, or causal regulation.

These additional signals offer candidate variables for models of transcript usage, post-transcriptional regulation, and perturbation response. Their predictive value must be tested rather than inferred from detection alone. Measurement-to-model studies should quantify assay bias and compare added RNA features with matched gene-count baselines through feature ablation, held-out evaluation, uncertainty calibration, and independent functional or perturbation tests.

The central advance from mRNA-focused profiling toward broader RNA measurement is therefore not simply a larger feature set. It is the opportunity to build experimentally testable models of RNA regulation and dynamic cellular response. Entity-specific digital twins remain a longer-term goal requiring longitudinal model updates and prospective validation, particularly in patient-level applications.

## Figures and Tables

**Table 1:** Representative single-cell transcriptomic technologies and their potential contributions to virtual-cell modeling.

Field	Technology	RNA Capture Spectrum	Transcript Coverage	Reported Cellular Scale	Potential Model Input	Reference
Low-throughput poly(A)-based RNA sequencing	Tang-seq	Mainly poly(A) RNA	Broad, uneven gene-body coverage	Individually processed cells	Cell-resolved gene expression	[10]
	Smart-seq/Smart-seq2	Poly(A) RNA, including polyadenylated lncRNAs	Broad, near-full-length gene-body coverage	Individually processed cells	Expression and splice features	[21,22]
	Smart-seq3	Mainly poly(A) RNA	Gene-body coverage with 5′ UMI counting	Individually processed cells	Molecule-linked expression and isoform features	[23]
	Smart-seq3xpress	Mainly poly(A) RNA	Broad gene-body reads with 5′ UMI counting	Individually processed cells; 26,260 cells across multiple 384-well plates	Gene, splicing and allelic features	[24]
High-throughput poly(A)-based RNA sequencing	Drop-seq/inDrop	Mainly poly(A) RNA	3′-end UMI counts	Droplet-scale; thousands of cells	Atlas-scale gene-count inputs	[11,25]
	Seq-Well	Mainly poly(A) RNA	3′-end UMI counts	3694 cells in a PBMC demonstration	Portable cell-state profiling	[26]
	10x Genomics GemCode	Mainly poly(A) RNA	3′-end UMI counts in the cited implementation	Droplet-scale; configuration-dependent	Atlas and foundation-model gene-count inputs	[12]
	sci-RNA-seq	Poly(A) RNA and captured nuclear pre-mRNA	Predominantly end-counted RNA	Nearly 50,000 cells in the cited study	Broad cell-state sampling	[27]
	SPLiT-seq	Poly(A) RNA; additional transcripts depend on priming strategy	Short-read, barcoded RNA profiles	156,049 nuclei in the cited study	Large-scale fixed-cell or nuclear profiles	[28]
Single-cell total RNA sequencing	SUPeR-seq	Poly(A), selected non-poly(A), and circular RNAs	Broad, uneven gene-body coverage	Individually processed embryonic cells	Candidate non-poly(A) and circular-RNA features	[29]
	MATQ-seq	Coding and non-coding total RNA	Broad, strand-aware coverage	Individually processed cells	Low-abundance and antisense RNA features	[30]
	RamDA-seq	Poly(A), non-poly(A), nascent, and enhancer RNAs	Broad gene-body coverage	Individually processed cells	RNA-processing and enhancer-RNA features	[31]
	Smart-seq-total	Coding RNAs, lncRNAs, and selected short RNAs	Broad RNA-size coverage; coverage bias remains	Hundreds of cells in the cited study	Coding and non-coding RNA features	[33]
	VASA-seq	Poly(A), non-poly(A), unspliced, and selected short RNAs	Broad, strand-specific gene-body coverage	Plate and droplet implementations; 33,662 cells in the cited analysis	Splicing and non-coding RNA features	[18]
	STORM-seq	Broad total-RNA spectrum, including selected regulatory and TE-derived transcripts	Broad paired-end gene-body coverage	Individually processed cells	Candidate regulatory-RNA and TE features	[34]
	TotalX	Coding, non-coding, and selected short RNAs	End-enriched total-RNA readout	301,515 cells using TotalX-miRNA(+) in the cited study	Scalable coding and non-coding RNA features	[35]
	dropTotal	Coding and non-coding total RNA, including lncRNAs and selected small RNAs	Broad, strand-specific gene-body coverage reported	Approximately 75,000 glioma nuclei in the cited study	Candidate ncRNA and splice-junction features requiring downstream validation	[36]
Long-read isoform sequencing	ScISOr-Seq	Predominantly poly(A)-derived cDNA from upstream capture	Cell-assigned long reads; isoform structure	Thousands of cerebellar cells in the cited study	Cell-type-specific isoform references	[37]
	R2C2	RNA spectrum determined by upstream cDNA preparation	Repeated-template nanopore consensus reads	Multiplexed cells; scale depends on upstream library	Consensus-supported transcript structures	[38]
	MAS-ISO-seq	RNA spectrum determined by upstream cDNA preparation	Concatenated cDNA with long-read isoform resolution	10x-derived cell-barcoded cDNA; cellular scale depends on upstream library	Cell-resolved isoform and splice features	[39]
	HIT-scISOseq	Inherits upstream 10x poly(A)-based capture	Cell-resolved long reads; isoform recovery depends on intact cDNA	3375 cells	Transcript isoform structures	[40]
Single-cell multi-omics	CITE-seq	Poly(A) RNA plus surface-protein tags	End-counted RNA and antibody-derived tags	Barcoded single cells; study-dependent	Paired RNA–protein states	[41]
	sci-CAR	Poly(A)-based RNA plus chromatin accessibility	RNA counts and accessible-chromatin features	Combinatorial indexing; thousands of cells	Paired RNA–chromatin features	[42]
	SHARE-seq	Poly(A)-based RNA plus chromatin accessibility	RNA counts and accessible-chromatin features	Split-pool indexing; study-dependent	Paired RNA–chromatin features	[43]
	TEA-seq	Poly(A)-based RNA, chromatin accessibility, and surface-protein tags	RNA, ATAC, and antibody-derived tags	29,264 quality-filtered cells in the cited analysis	Paired transcriptional, chromatin, and protein states	[44]

The RNA capture spectrum lists RNA classes reported as detectable and does not imply equivalent recovery efficiency across classes. Transcript coverage and sensitivity depend on sample preparation, sequencing depth, and analysis. Reported cellular scale describes the cited demonstration or analysis, which may pool samples or experiments; it does not indicate per-run capacity or a depth- and budget-matched throughput ranking. Long-read rows describe structural resolution of molecules recovered by upstream capture, not broader RNA-biotype capture. The cited STORM-seq [34] and dropTotal [36] studies are preprints. The dropTotal comparisons are developer-reported and should not be interpreted as independently validated performance rankings. Potential model inputs do not imply demonstrated improvements in virtual-cell prediction. Abbreviations: ATAC, assay for transposase-accessible chromatin; lncRNA, long non-coding RNA; ncRNA, non-coding RNA; PBMC, peripheral blood mononuclear cell; TE, transposable element; UMI, unique molecular identifier; cDNA, complementary DNA; miRNA, microRNA; mRNA, messenger RNA.

**Table 2:** Modeling layers of virtual cells based on single-cell omics.

Layer	Representative Models	Major Capabilities	Typical Inputs	Key Limitations	Reference
Latent cell-state representation	scVI, scANVI, totalVI	State embedding, integration, denoising and joint RNA–protein modeling	Gene counts, batch/biological covariates and antibody-derived tags	Descriptive focus; limited directionality and causality	[13,45,46]
Gene regulatory networks	SCENIC, SCENIC+, CellOracle	Regulon and enhancer–gene inference; TF-perturbation simulation	Gene expression and TF motifs; optional ATAC and trajectory data	Correlation- and prior-dependent; limited post-transcriptional coverage	[47,48,49]
Cell-state dynamics	RNA velocity, Dynamo, CellRank, Waddington-OT	Transition direction, vector fields, fate probabilities and cross-time mapping	Spliced/unspliced RNA, metabolic labeling or time-series data	Kinetic or transport assumptions; sampling sensitivity	[15,50,51,52,53]
Perturbation-response prediction	CPA, GEARS	Drug, genetic and combinatorial perturbation prediction	Perturb-seq, perturbation labels, doses/covariates and gene graphs	Limited training coverage; weak combinatorial extrapolation; mRNA-centered outputs	[54,55]
Single-cell foundation models	Geneformer, scGPT, scFoundation, CellPLM, UCE, CellFM	General representations, annotation, integration, transfer and perturbation prediction	Large expression corpora; optional tissue, multi-omic or perturbation context	Corpus and platform biases; objective mismatch; unstable zero-shot generalization	[16,17,57,58,59,60,61]

The layers indicate the principal functional roles of the models rather than mutually exclusive categories. Individual models may span representation learning, network inference, and perturbation prediction. ATAC, assay for transposase-accessible chromatin; TF, transcription factor; OT, optimal transport.

**Table 3:** Assay inputs and validation evidence for representative virtual-cell models.

Model	Actual Input	Single-Cell Total-RNA Input?	Original Benchmark	Reported Evaluation	Uncertainty Assessment	Key Limitation	Reference
scVI	scRNA counts; batch	No	Integration; differential expression	Held-out-cell likelihood; batch-integration benchmarks; OOD holdout: NR	Posterior; calibration NR	Associational latent space	[13]
scANVI	scRNA counts; labels	No	Annotation; integration	Partial-label annotation and cross-dataset tests; biological-condition OOD holdout: NR	Label posterior; calibration NR	Reference-label dependence	[45]
totalVI	CITE-seq RNA; protein tags	No	Integration; protein imputation	Held-out measurements; batches	Held-out RNA/protein count calibration assessed; perturbation-prediction calibration: NR	Antibody-panel limited	[46]
SCENIC	scRNA counts; TF motifs	No	Regulons; clustering	Agreement with published cell-type labels; OOD holdout: NR	NR	Links are not causal proof	[47]
SCENIC+	scRNA; scATAC; motifs	No	Enhancer regulons	Comparisons with ChIP-seq and Hi-C data; perturbation analyses; biological-condition OOD holdout: NR	NR	Enhancer links are indirect	[48]
CellOracle	scRNA; accessibility-based GRN	No	TF-perturbation simulation	Zebrafish TF-loss experiments	Calibration NR	Prior-GRN dependence	[49]
RNA velocity/scVelo	Spliced & unspliced counts	No	Transition direction; kinetics	Developmental-lineage and cross-dataset evaluations; OOD holdout: NR	Calibration NR	Kinetic and coverage bias	[15,50]
Dynamo	Splicing; labeled new/old RNA	No	Rates; vector fields	Applications across metabolically labeled datasets; OOD holdout: NR	NR	Assumes sufficient state variables	[51]
CellRank	Expression graph; velocity	No	Fate probabilities	Lineage tracing; lung experiments	Velocity uncertainty propagated	Inherits velocity bias	[52]
CPA	Perturbation scRNA; dose; context	No	Response prediction	Unseen dose/context combinations	Distance-based score	Limited for novel perturbations	[54]
GEARS	Perturb-seq counts; gene graphs	No	Single-/multigene responses	Unseen targets and combinations	Predicted variance; calibration NR	Graph/context dependence	[55]
Geneformer	Ranked scRNA gene profiles	No	Network tasks; target discovery	Task splits; cardiac experiments	NR	Omits transcript structure	[16]
scGPT	Gene tokens; expression values	No	Annotation; perturbation	Query cells; held-out perturbations	NR	Gene-count input	[17]
scFoundation	Gene counts	No	Denoising; annotation; response	Task-specific splits	NR	Downstream-model dependence	[57]
CellPLM	scRNA; spatial neighborhoods	No	Clustering; spatial imputation	Datasets absent from pretraining	NR	Neighborhood/assay dependence	[58]
UCE	scRNA-seq gene counts; ESM2-derived protein-sequence embeddings for gene tokens	No	Zero-shot cell embedding	Unseen datasets/species	NR	Protein-coding focus	[59]
CellFM	Predominantly gene counts	No	Annotation; perturbation	Inter-dataset tests; perturbations	NR	10x-heavy training corpus	[60]

*No* indicates that the cited study did not report single-cell total-RNA sequencing as a model input; this designation concerns assay capture chemistry, not the size of the gene-count matrix. *NR* means that the specified assessment was not reported. An out-of-distribution (OOD) holdout here refers to a prespecified test on an unseen donor, tissue, platform, or perturbation. Held-out cells or measurements, comparisons with published annotations, cross-dataset applications, and independent experimental validation provide different forms of evidence and should not be treated as interchangeable. The ESM2-derived protein-sequence embeddings used by UCE are gene-token features, not protein abundance measured in the profiled cells. Model-derived posteriors or uncertainty scores do not, by themselves, establish calibrated predictive uncertainty.

**Table 4:** RNA information layers and evidence for virtual-cell modeling.

Information Layer	Demonstrated Observables	Proposed Model Use	Required Validation	Evidence Status	Reference
Transcript resolution	Junctions; isoforms; poly(A) sites	Transcript-level states	Held-out gain vs. gene counts	Observed; model gain untested	[18,62,68]
Non-coding RNA features	lncRNAs; selected small RNAs; locus-resolved TE-derived transcripts	Candidate regulatory features	Perturbation; orthogonal assays	Detection established; causal regulatory roles require targeted validation	[18,35,67,69]
RNA kinetics	Unspliced; labeled RNA	Kinetic models	Time-course; turnover assays	Signals measured; rates inferred	[15,18,50,51,70,71,72,73]
Model vocabulary	Transcript and RNA events	Event-level pretraining	Ablation; held-out tests	Proposed	[16,17,57,60,62]
Perturbation response	Gene and APA shifts	Intermediate-response models	Unseen perturbations; phenotype	Responses shown; model gain untested	[14,74]
Spatial context	RNA localization; spatial maps	Spatial conditioning	Imaging; spatial intervention	Observed in separate assays	[76,77]
Measurement reliability	Recovery; mapping; depth	Assay-aware models	Spike-ins; cross-platform tests	Bias documented; model gain untested	[20,35,69,78,79]

Observables may come from different assays; no single total-RNA method measures every layer. Detection alone does not establish mechanism. Proposed model gains require matched gene-count baselines and held-out validation. Abbreviations: lncRNA, long non-coding RNA; TE, transposable element; APA, alternative polyadenylation.

**Table 5:** Proposed matched measurement-to-model benchmark for testing the added value of broader single-cell RNA measurements.

Component	Proposed Design	Purpose
Samples	Matched aliquots; randomized processing; cell-composition checks	Control biological and batch variation
Measurement	Poly(A)-based vs. broader-RNA assays; equal-read and equal-budget comparisons; report cell recovery, estimated RNA-molecule recovery and informative non-rRNA depth	Separate signal gain from resource use
Features	Gene-only baselines; add ncRNA, splice and isoform features incrementally	Isolate each feature layer
Models	Match architecture, training and preprocessing where feasible; include simple baselines	Limit model confounding
Generalization	Hold out donors, tissues, platforms or perturbations	Test transfer
Endpoints	Feature reproducibility, prediction, calibration and functional validation	Assess biological utility
Interpretation	Report effect sizes, uncertainty, failed tasks and cost per gain	Avoid universal assay rankings

This is a proposed benchmark, not an experiment performed in this review. Matched aliquots are not the same physical cells. Equal-read and equal-budget comparisons are separate analyses; kinetic features require additional temporal measurements. Abbreviation: ncRNA, non-coding RNA.

## Data Availability

No new data were created or analyzed in this study. Data sharing is not applicable to this article.
